# Acute insertion effects of penetrating cortical microelectrodes imaged with quantitative optical coherence angiography

**DOI:** 10.1117/1.NPh.3.2.025002

**Published:** 2016-04-19

**Authors:** Daniel X. Hammer, Andrea Lozzi, Adam Boretsky, Cristin G. Welle

**Affiliations:** a Center for Devices and Radiological Health, Food and Drug Administration, Division of Biomedical Physics, Office of Science and Engineering Laboratories, 20903 New Hampshire Avenue, Silver Spring, Maryland 20993, United States; b University of Colorado Denver, Departments of Neurosurgery and Bioengineering, 12700 East 19th Avenue, Aurora, Colorado 80045, United States

**Keywords:** optical coherence tomography, angiography, neurophotonics, neurotoxicity, inflammation, cranial window

## Abstract

The vascular response during cortical microelectrode insertion was measured with amplitude decorrelation-based quantitative optical coherence angiography (OCA). Four different shank-style microelectrode configurations were inserted in murine motor cortex beneath a surgically implanted window in discrete steps while OCA images were collected and processed for angiography and flowmetry. Quantitative measurements included tissue displacement (measured by optical flow), perfused capillary density, and capillary flow velocity. The primary effect of insertion was mechanical perturbation, the effects of which included tissue displacement, arteriolar rupture, and compression of a branch of the anterior cerebral artery causing a global decrease in flow. Other effects observed included local flow drop-out in the region immediately surrounding the microelectrode. The mean basal capillary network velocity for all animals was 0.23 (±0.05  SD) and 0.18 (±0.07  SD) mm/s for capillaries from 100 to 300  μm and 300 to 500  μm, respectively. Upon insertion, the 2-shank electrode arrays caused a decrease in capillary flow density and velocity, while the results from other configurations were not different from controls. The proximity to large vessels appears to play a larger role than the array configuration. These results can guide neurosurgeons and electrode designers to minimize trauma and ischemia during microelectrode insertion.

## 1 Introduction

Cortical electrodes are increasingly used to treat clinical neurological disorders (e.g., Parkinson’s disease, epilepsy) with stimulation or combined recording and stimulation, as well as for neuroprosthetic control in brain–computer interface (BCI) devices.^1^^–^^3^ Although surface electrodes are a viable option for some applications,^4^ penetrating microelectrodes have access to neurons throughout the cortical column and thus enable more complete neural sampling for BCI applications that require higher fidelity neural sensing, control, and tuning. Neural electrode signal degradation can accompany chronic use and may ultimately limit the effectiveness of neural devices that use penetrating electrodes.^5^ Much information on the neuroinflammatory response to implanted electrodes has been acquired from postmortem histology.^6^^–^^8^ Chronic device implantation has also been characterized with an *in vitro* simulated reactive accelerated aging environment.^9^ Despite new methodologies to provide histological characterization of intact tissue surrounding implanted cortical microdevices,^10^ several issues confound interpretation of results, including inability to track response dynamics in a single animal in the presence of large subject variability and processing artifacts.

High-resolution optical imaging can provide a clear picture of some of the cellular responses to microelectrode implantation in tissue. In neuroscience, two-photon microscopy (TPM) has become a relatively mature tool to explore tissue architecture and dynamics,^11^ both in normal adaptations such as learning,^12^ but also in diseases such as stroke.^13^ In combination with genetic models that express fluorescent biomarkers, TPM is able to image neuronal and non-neuronal support cells.^14^^,^^15^ Flowing blood can be imaged with TPM using exogenous dyes.^16^ Another optical imaging modality, optical coherence tomography (OCT), has begun to supplement TPM for neuroscience applications, particularly in angiography, where intrinsic contrast techniques are able to resolve cortical vasculature,^17^ quantify flow,^18^ quantify cortical oxygen metabolism,^19^ and even resolve neuronal cell bodies.^20^ Amplitude decorrelation (or speckle variance) OCT techniques detect flowing blood cells and distinguish their dynamic signal from the static light scattering component. This capability, particularly when coupled with TPM,^21^ can provide significant information on neurovascular hemodynamics and neuronal energy demands. Recent quantitative optical coherence angiography (qOCA) imaging of acute and permanent stroke mouse models captured and quantified flow in regions of occlusion and reperfusion and well as longer term capillary remodeling.^22^ qOCA has also been used to describe the significant role of penetrating arterioles in the regulation of blood flow in response to focal stroke.^23^

Postmortem histological studies have provided a picture of the acute cellular response to electrode implantation: release of proinflammatory cytokines and immediate microglial reactivity, followed by neurodegeneration in the surrounding region and the formation of a tight glial capsule immediately around the electrode.^24^^,^^25^ The vascular response includes capillary proliferation^25^ and disruption of the integrity of the blood-brain barrier.^26^ Using immunohistochemistry to label neurons, microglia, and astrocytes, Knaack et al.^27^ have shown distinct differences in the tissue response depending upon electrode type.

The cellular reactions to implanted microelectrodes have also been observed with *in vivo* optical imaging. Using TPM, Kozai et al.^28^ found increased microglial activity—extending processes toward the electrode—but little cell body migration in the first 6 h after implantation. However, there was also evidence of vascular displacement and possibly capillary rupture in that study. Our own initial long-term investigation of single-shank penetrating microelectrodes using OCT found significant angiogenesis and vascular remapping in the superficial layers under an implanted window, but minimal deeper capillary remodeling in the region around an electrode over the course of the first few months after implantation.^29^ Combined OCT and TPM imaging of the same region around implanted 2- and 4-shank electrodes revealed significant neuronal and vascular disruption in the weeks following insertion.^21^

Building upon our earlier work^21^^,^^27^^,^^29^ and the findings of Kozai et al.,^28^ the aim of this study was to look for vascular changes during microelectrode insertion. We designed an experiment to collect OCT angiograms and flow maps while a penetrating microelectrode was inserted into mouse cortical tissue in discrete steps. This experimental design could reveal any acute changes, as well as the initiation of neuroinflammatory events whose time-course extends through multiple phases over months. During electrode insertion, we observed significant tissue displacement in all animals, accompanied by both local and global changes to the capillary network in some animals. We did not observe repeatable capillary remapping, density, or flow changes associated with electrode insertion in any animals. These findings contribute to a complex picture of tissue reactivity, where hypoxia can be induced immediately, but the predominant neuroinflammatory response and vascular remodeling due to electrode insertion take place on longer time-scales of weeks or months after implantation.

## 2 Materials and Methods

### 2.1 Animals and Surgery

**Fig. 1:**
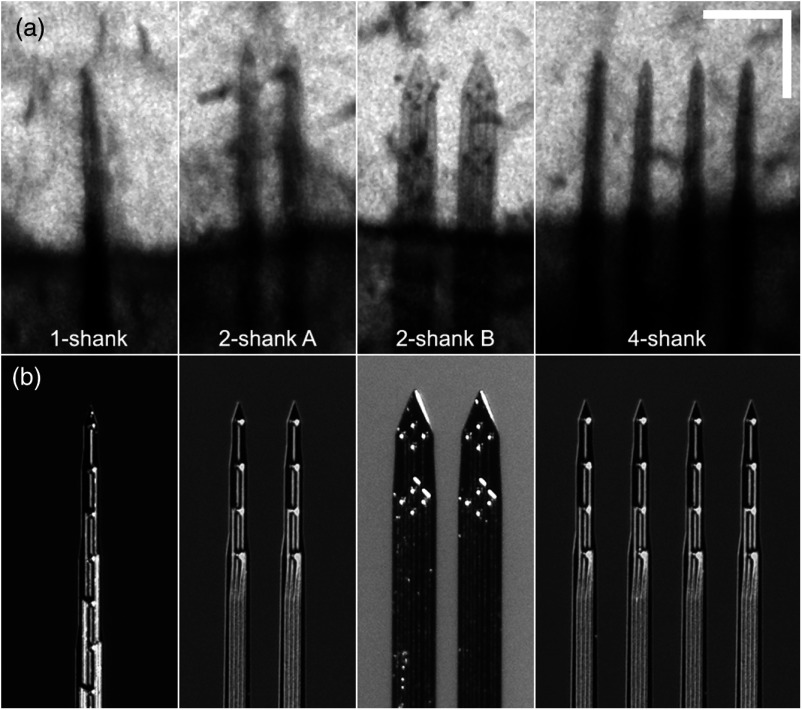
(a) OCT reflectance images and (b) surgical microscope photographs of four electrode configurations tested. Individual recording sites and traces can be resolved in OCT image of the 2-shank B electrode. Scale bar=200  μm. (W79, W84, W78, W76).

**Fig. 2:**
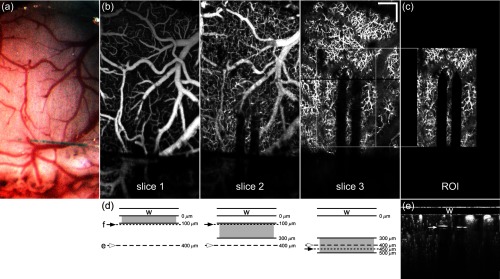
Depth sectioning with optical coherence angiography. (a) Photograph of cortical preparation and window through surgical microscope. (b) Three integrated depth projections, slice 1: 0 to 100  μm, slice 2: 100 to 300  μm, and slice 3: 300 to 500  μm below implanted window. (c) 0.6×1.0  mm (300×500-pixel) ROI with electrode exclusion used for analysis. (d) Diagram of integrated depth regions corresponding to the window (w), approximate focus plane (f), and approximate location of electrode tips (e) after the third insertion step. (e) Composite cross-sectional OCA image created by integrating two focal planes corresponding to position shown in slice 3 (black line) and indicating electrode tip position (white arrow). Scale bar=200  μm. (W78).

All procedures were approved by the Food and Drug Administration (FDA) White Oak Institutional Animal Care and Use Committee. A total of 16 wild-type mice (C57BL/6J) were used in the study, 12 of which had cortical microelectrodes inserted (Fig. 1) and four of which were used as controls, which had craniotomy and window implantation but no cortical electrodes inserted. Animal numbers are indicated in figure captions and text by WXX, where XX is the animal number. The mice were anesthetized with isoflurane (4% induction, 1.5% maintenance, 0.8  L/min $O_{2}$) and their body temperature was maintained at 37 deg with a heating pad during surgery and imaging. A craniotomy was performed over the motor cortex, exposing a region roughly $2\times 3\;{\mathrm{mm}}^{2}$. A small glass cover slip was gently placed over the cortex and affixed in place on three sides with cyanoacrylate glue [Fig. 2(a)]. A metal head-bar was also secured to the contralateral skull to prevent head motion during imaging. Because the posterior side of the preparation was left exposed for electrode insertion, the preparation was irrigated frequently with sterile saline during the procedure.

### 2.2 Electrodes

1-, 2-, and 4-shank Michigan-style silicon microelectrodes (NeuroNexus, Ann Arbor, Michigan) were inserted into three animals each. For the 2-shank electrode, two electrode types with different diameters were inserted into three animals each. Figure 1 shows OCT reflectance images of the four electrode configurations. The shanks measured 50  μm (1-, 2A-, and 4-shank) and 95  μm (2B-shank) in width (near the tip), 3 mm in length, and 15  μm in thickness. For multiple shank microelectrodes, the spacing between shanks was 100  μm. In addition, a control group without inserted electrodes was also imaged at the same time-points as the animals with inserted electrodes. The microelectrodes were inserted in three or four steps and imaged with ∼250  μm linear insertion distance for each step (step distance was adjusted slightly to avoid shadowing from larger vessels). The targeted depth of the electrode tip after the final insertion step was 400  μm. The actual depth measured from the OCT volumes was 358  μm (SD=101  μm) for the 12 electrode animals, where the depth of the deepest shank was used for multishank electrodes. Each insertion step took ∼5 to 10 s for an insertion speed of roughly 25 to 50  μm/s. Imaging for each step took ∼30  min and so the entire session lasted 2 to 2.5 h. The 2B-shank electrodes are arranged in a tetrode configuration with two tetrode arrays per shank (Fig. 1). The 1, 2A-, and 4-shank microelectrodes each had 16 recording sites, though electrophysiological recordings were not collected during the insertion study. The microelectrodes were inserted at an angle of ∼14 to 17 deg with respect to the cortical surface.

### 2.3 Imaging System

A spectral domain OCT system was used for imaging and has been described previously.^29^ The system uses a superluminescent diode at a wavelength of 1.3  μm and bandwidth of 85 nm (Exalos, Switzerland). A custom spectrometer (Wasatch Photonics, Durham, North Carolina) matched to the source collected the OCT spectra. The maximum line rate of the InGaAs linear detector (UTC Aerospace Systems/Sensors Unlimited, Princeton, New Jersey) is 76 kHz. A 5× magnification telecentric scan lens (ThorLabs, Newton, New Jersey) was used to the focus the illumination light on the mouse motor cortex. The system was characterized with a nanoparticle-embedded phantom and the axial and lateral point spread function was found to be 9.4 and 6.5  μm, respectively. All angiography processing was performed in real-time on the video card graphical processing unit (GPU, NVIDIA GeForce GTX-760 video card) using custom parallel computing OCA processing using compute unified device architecture (CUDA) code. The custom OCT acquisition software, which handled all the hardware (galvanometers, framegrabber), timing, front-end signal conditioning, and interface to the GPU libraries, was written in LabVIEW (National Instruments, Austin, Texas).

The animal was positioned on a three-axis animal stage to precisely position the window in the field of view of the scanners. Stereotaxic stages (Narishigi, East Meadow, New York) were used to insert the microelectrode into the mouse cortex. The stereotaxic stage was mounted to the animal stage.

### 2.4 Imaging Protocol

Prior to electrode insertion, larger-field baseline scans ($2\times 2\;{\mathrm{mm}}^{2}$, 500×500 lateral pixels, excluding fly-back) were acquired of the entire window at two focus depths separated by 250  μm in air (∼350  μm in tissue, see Fig. 2). These scans were used to guide initial electrode alignment to avoid large vessels during insertion. The two focus depths covered the entire depth range of insertion, with the second depth chosen to align to the final position of the electrode tips after the last insertion step. At baseline and at each electrode insertion step, four strip scans ($1\times 2\;{\mathrm{mm}}^{2}$) were acquired, two at each of the two focus depths. The strip scans were set so that the long axis was parallel to the electrode (Fig. 2). The first set (250×500 lateral pixels) was acquired with a gate length (number of B-scans acquired at each lateral position) of 100 for capillary velocimetry. The second set (500×1000 lateral pixels) was acquired with higher pixel density and gate length of 10 for angiography. Owing to the high gate length for the flow set and the high pixel density for the angiography set, the four scans each took about 7 min to acquire and process on the GPU.

### 2.5 Angiography and Capillary Velocimetry Processing

The OCT datasets were processed in a customary manner with background subtraction, λ-k resampling, Fourier transformation, and logarithmic scaling to produce reflectance sequences (videos).^30^ Angiography sequences were produced using the average absolute difference of 10 and 100 adjacent B-scans with an interval of 7.46 or 4.16 ms.^31^

Flow sequences were produced using an algorithm for capillary velocimetry modeled after one proposed by Srinivasan et al.^32^ and others,^33^^,^^34^ which estimates the capillary flow velocity from the calculated width of the power spectral density (PSD) of the dynamic scattering component of the OCT signal. The algorithm calculates the autocorrelation function of the four-dimensional complex OCT signal, summed over a gate length defined by the number of adjacent B-scans collected. The correlation width, which defines the spectral resolution with which the PSD is sampled, was set to eight. The estimated PSD is calculated by discrete Fourier transformation of the absolute value of the autocorrelation function, and the frequency bandwidth (Δf) calculated from the sum of the normalized PSD frequencies. The Δf map was smoothed with a sigmoid transparency, the width set for the B-scan interval, and the threshold set empirically according to flow range (0.0 to 0.8  mm/s). Three OCT volumetric sequences (reflectance, angiography, and flow) were created for each scan at each depth for every insertion step of each animal. Except for the sigmoid transparency, all processing was performed on the GPU during imaging.

The dynamic range of the measurable flow velocity is bounded on the lower end (threshold) by system noise sources and on the upper end (saturation) by the degree of decorrelation for techniques that rely on complex decorrelation such as the one used in this study. Between threshold and saturation, the flow velocity is linear and the relationship between Δf and velocity can be calibrated in a straightforward manner. We used various diameter flow cells to verify a parabolic (nonturbulent laminar) flow profile and a uniform scattering phantom (Spectralon, Labsphere Inc., North Sutton, New Hampshire) on a precision linear stage moving at constant velocity for calibration. The accuracy of the precision linear stages was also verified with a grid phantom (Arden Photonics Ltd., United Kingdom) using high frame rate OCT imaging. Further flow quantification analysis (described below) used only data taken within the linear calibration range from 0.0 to 0.8  mm/s, which generally includes flow in the capillaries but not the larger vessels.

### 2.6 Analysis

Integrated depth projections were created from the processed volumetric OCA scans. As shown in Fig. 2(b), the depth projections were summed over three depths relative to the bottom of the window: 0 to 100  μm (slice 1), 100 to 300  μm (slice 2), and 300 to 500  μm (slice 3). Three depth slices were used to span the depth through which the electrode passes over the course of the three (angled) insertion steps, but also to observe, quantify, and control for inflammation related to craniotomy and window insertion surgery. For reflectance images, average-intensity projection (AIP) was always used. For angiograms, AIP was used for slice 1 and 2 and maximum-intensity projection (MIP) was used for slice 3. For flow maps, MIP was used for all slices (to calculate the maximum flow in the measured column). Each set of depth projections for baseline and all insertion steps were then registered with StackReg (Fiji). The largest lateral and axial shift occurred between the baseline and first insertion step owing to tissue movement during initial insertion.

After registration, the depth projections were processed further using three custom programs written in LabVIEW to quantify various insertion effects. In all three programs, a region-of-interest (ROI) was chosen to confine analysis to the region immediately adjacent to the electrode. A square or rectangular ROI was manually selected to be centered with respect to the insertion point [Fig. 2(c)]. The image pixels associated with the electrode itself were manually excluded (using a polygon selection tool) using the reflectance image of the deepest insertion step (usually step 3). For the control animals, an ROI in the center of the window was selected with no exclusion zone. This ensured that electrode artifacts were excluded or minimized, and the analysis was performed on the same number of pixels for baseline and all steps. Because the microelectrode size was different between groups and insertion depth different for every animal, the analysis was necessarily performed with a different number of pixels analyzed, but was referenced to baseline or normalized to the ROI size to prevent measurement bias related to the ROI size.

For analysis of mechanical tissue displacement, a $0.6\times 1.0\;{\mathrm{mm}}^{2}$ (300×500-pixel) ROI in the most superficial slice (slice 1) of the angiogram was measured using optical flow. Perfused capillary density was measured in all three slices of the angiogram using the same ROI as used for optical flow. Flow velocity was measured in a $0.6\times 1.0\;{\mathrm{mm}}^{2}$ (150×250-pixel) ROI in the deeper two slices (slices 2 and 3) of the flow maps. The ROIs for the optical flow, perfused capillary density, and flow maps were typically identical regions (but with different pixel density). Further detail on the analysis follows.

Optical flow is a frequently used processing technique for image registration or to characterize local motion within sets of images.^35^ To quantify the extent of mechanical tissue displacement during electrode insertion, the optical flow was calculated between sequential sets of angiograms, from baseline to first step, first step to second step, and so on. Processing sequential images gave a measure of tissue displacement between electrode insertion steps, while referencing the image to the baseline image yields cumulative displacement. The total displacement of the insertion was calculated from the optical flow measured from baseline to the final image. The program written to quantify mechanical displacement used an optical flow subroutine based upon the Lucas and Kanade algorithm.^36^ The window size for the optical flow calculation was 15×15  pixels. Preprocessing with registration ensured that only relative, local displacements arising from electrode insertion were included in the optical flow calculation. The optical flow was calculated only in the most superficial slice (0 to 100  μm), where the larger vessels were in best focus (for the first focus depth) and reflection artifacts related to the electrode were minimal.

To quantify changes in the capillary network, the perfused capillary density was measured in a similar manner as performed in our previous study^29^ using the angiograms from all slices. Speckle variance OCA is sensitive to flowing erythrocytes, such that a signal decrease is indicative of decreased flow and not necessarily a structural change. Perfused capillary density is used in this paper to indicate cross-sectional flow changes, as distinct from the actual capillary flow changes described later. Perfused capillary density was quantified in a slice as the fractional area of pixels assigned to vessels via thresholding divided by the total number of ROI pixels. An absolute threshold rather than a local adaptive threshold provided the best delineation of capillaries for these depth projections with relatively uniform brightness, and a particle filter (up to five pixels) was used to remove a very small amount of image noise. Large vessels were not particularly avoided, though the analysis was referenced to baseline so thresholding biases and those associated with differences between animals were minimized. The $\text{percent change}=\Delta a/a_{0}$, where a is the fractional area and $a_{0}$ is the baseline fractional area before insertion.

There are several possible causes for changes in capillary visibility. Observed changes in the capillary network associated with electrode insertion in any integrated slice may be associated with: mechanical displacement, which can move vessels into or out of focus or otherwise cause vessel distortion (both physically and optically); flow deficits, which can be caused by compression, partial occlusion, or some other vasoconstriction mechanism; increases in flow caused by vasodilation; shadowing of capillaries below the electrode or other larger vessels; and other artifactual effects that change capillary visibility (surface debris, and so on). These effects can be local or global in nature, depending on the physiological state and mechanical perturbations.

Changes in capillary velocity were quantified using the calculated flow maps with sigmoid transparency. Because the larger superficial vessels were largely outside of the linear flow measurement range (0.0 to 0.8  mm/s), the analysis was applied to slices 2 and 3 but not slice 1. For pixels within the ROI with electrode exclusion of the calculated flow map, the pixel value was converted to flow velocity using the slope and intercept of the calibration curve, excluding from analysis any pixels outside of the linear flow measurement range. The mean flow velocity (v) is calculated from all pixels within the ROI, and the total flow (f) is defined as the total ROI pixel count within the range, accounting for ROI size. The percent change for mean velocity and total flow were calculated in the same manner as the factional area ($\Delta v/v_{0}$ and $\Delta f/f_{0}$).

## 3 Results

### 3.1 Control Group Angiography

**Fig. 3:**
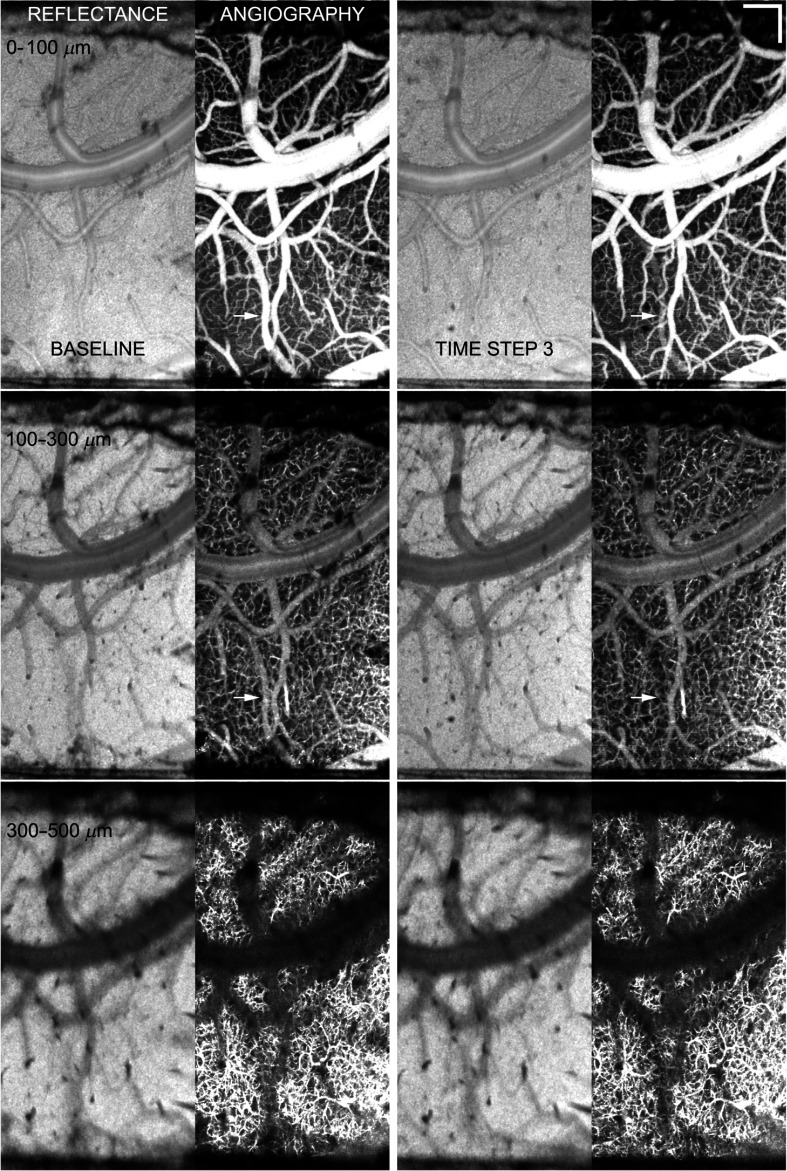
Example of control animal with little observable change in cortical vasculature beneath window in acute phase. Shown are sets of OCT reflectance and angiography images at three depths (0 to 100  μm, 100 to 300  μm, and 300 to 500  μm) for baseline and the third time step (∼1.5  h after baseline). All reflectance and angiography images are AIP except the third depth angiography, which is MIP. No vessel dilation or increase in perfused capillary density is observed. White arrow indicates one medium-sized superficial vessel that showed transient flow drop-out (time step 3) thought to be associated with normal physiological flow variability. Scale bar=200  μm. Video 1 shows all layers and time steps. (Video 1, AVI, W88, 1 MB) [URL: https://doi.org/10.1117/1.NPh.3.2.025002.1].

**Fig. 4:**
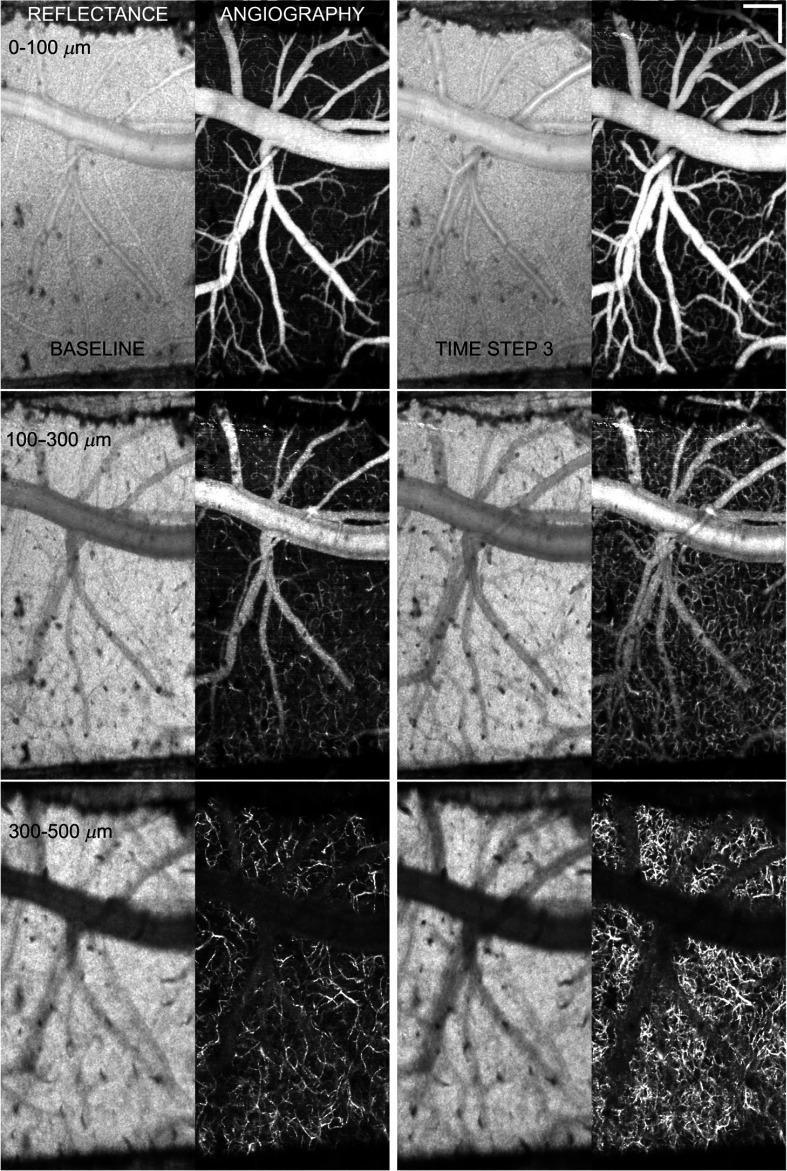
Example of control animal with vessel dilation and increase in perfused capillary density beneath window in acute phase. Panel layout is identical to Fig. 3. Scale bar=200  μm. Video 2 shows all layers and time steps. (Video 2, AVI, W87, 1 MB) [URL: https://doi.org/10.1117/1.NPh.3.2.025002.2].

In order to quantify vessel and flow changes that occurred as a result of microelectrode insertion, angiograms and flow maps from the four control animals were used to quantify acute changes that resulted from anesthesia, craniotomy, and window placement alone. In two control animals, there were no observed changes in angiography or blood flow at any time step. Figure 3 shows the reflectance images and angiograms for all three depths for baseline and after the third insertion time step for one of these two. The two sets of images appear qualitatively similar in terms of capillary pattern, density, and medium to large vessel diameter. In this animal, there was one medium-sized superficial vessel (Fig. 3, white arrow) that exhibited transient flow drop-out and reperfusion. This is frequently observed, though typically in smaller capillaries, and is attributed to normal physiological variability in blood flow. In the other two control animals, there was a marked increase in the angiography and flow signals. Figure 4 shows the results of the same analyses as Fig. 3 for one of the control animals with increased blood flow. In this animal, there was a noticeable increase in perfused capillary density, particularly in the deepest slice, accompanied by vasodilation in the larger vessels supplying the capillaries. No control animals exhibited a decrease in perfused capillary density or flow. These examples demonstrate the observed variability in global perfused capillary density and blood flow over time and between animals.

### 3.2 Mechanical Tissue Perturbation

**Fig. 5:**
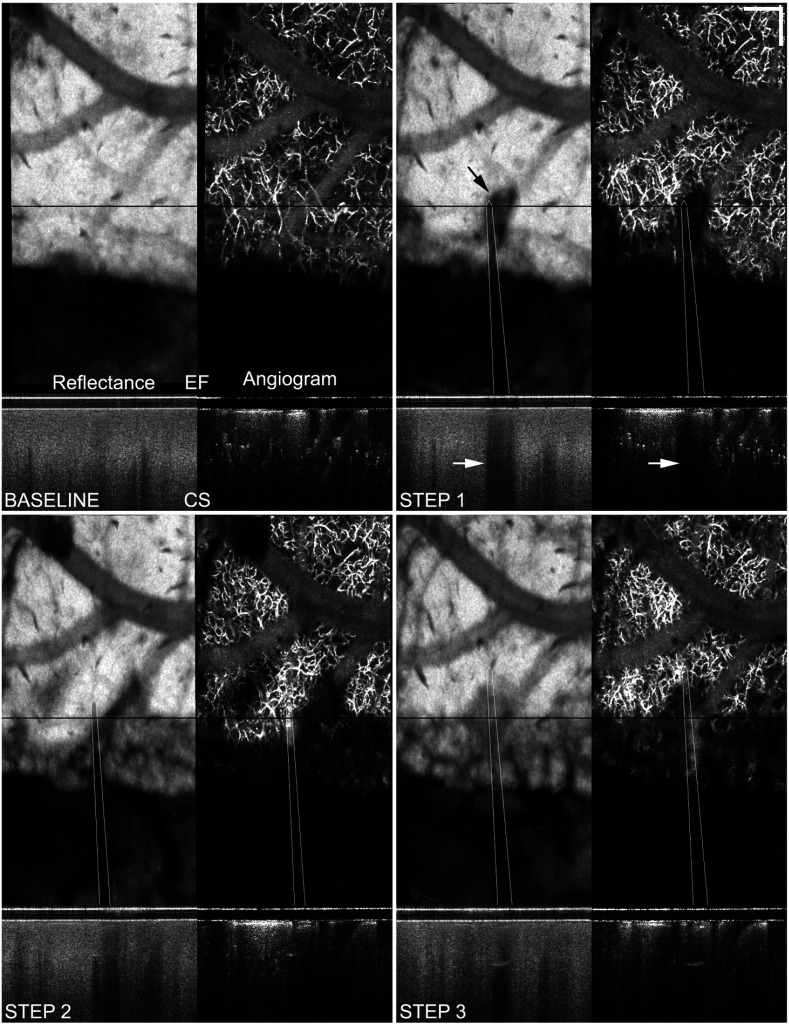
Vessel is nicked during single-shank electrode insertion (step 1, black arrow) causing superficial bleeding. *En face* (EF) and cross-sectional (CS) OCT reflectance images and angiograms are shown for each step. Location of corresponding CS image is indicated on EF image by black line. Approximate electrode location is also indicated with overlay. Pooled blood causes a distinct shadow region initially (white arrows), diffuses into the interstitial space, and does not completely clear in subsequent steps. Extravasation causes scattering and absorption which blocks penetration of the OCT signal. Note especially the loss of capillary contrast in the CS angiogram in step 3. Depth slice 3 (300 to 500  μm) is shown. Scale bar=200  μm (W80).

The most obvious and immediate mechanical effect of electrode insertion is puncturing a vessel. The craniotomy surgery itself can be traumatic and cause some damage to superficial vessels resulting in bleeding. In this study, nearly all animals (14 of 16) began with surgical preparations absent any vessel damage or sign of extravasation. The other two animals had minimal bleeding that, for the most part, resolved before imaging. Image-guided electrode insertion into the cortical tissue below a window mitigated the risk of puncturing large vessels. In one study animal, a medium-sized vessel was nicked during insertion, causing extravasation as shown in Fig. 5. Large vessels were successfully avoided in all animals, and no extravasation was visible from small vessels or capillaries.

**Fig. 6:**
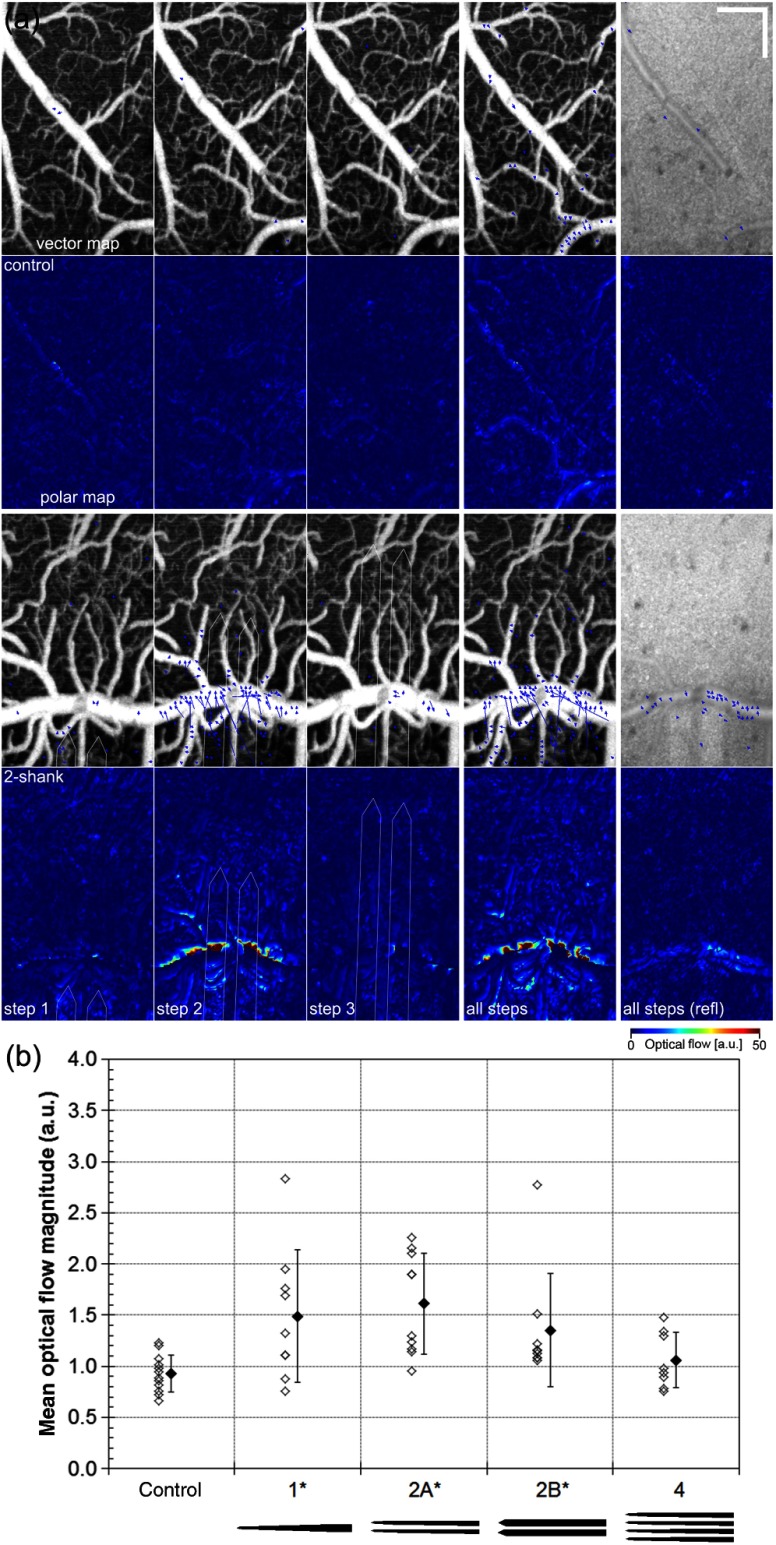
Optical flow of angiogram measures mechanical perturbation. (a) Example vector (overlaid on angiogram) and polar (magnitude) maps for control (top two rows) and 2-shank electrode insertion (third and fourth rows) for individual steps and all steps (baseline to last step) for depth slice 1 (0 to 100  μm). Shown for comparison are the vector and polar maps for the corresponding AIP reflectance images for all steps. Scale bar=200  μm. (b) Mean optical flow velocity magnitude across the ROI for all animals. Open symbols are values for each step in the animal group. Closed symbols are the mean values for the group. Error bars represent ±SD. *p<0.05 between control and electrode groups. Electrode footprints are shown below graph (W74 and W87).

The visualization of the fine capillary network via OCT angiograms allowed measurement of the total lateral tissue displacement upon electrode insertion, which in some cases was ∼35  μm. The optical flow was used to quantify local tissue motion, where vector and polar flow maps were created between sequential sets of angiograms as described earlier. Axial displacement was constrained by the glass window affixed to the edges of the craniotomy, and was not measured in this analysis. The polar maps illustrate the displacement magnitude, whereas the vector maps indicate the magnitude and direction. The vector maps display a sparse, thresholded representation of the displacement field, where the threshold = 5 and the step size = 10. Figure 6 shows the results from one control animal and one animal with a large displacement upon 2-shank electrode insertion. In the control animal, only minimal displacement is observed, partially from vasodilation of arterioles. In the 2-shank electrode animal, the displacements were large, particularly for insertion step 2, where the electrode passes near a large horizontal anterior cerebral artery (ACA) branch. For comparison, the optical flow was calculated on the reflectance images [last column in Fig. 6(a)], which yielded significantly lower displacement values because of lack of contrast. Though not strictly necessary, the additional contrast from the capillary network clearly enhanced the sensitivity (and possibly also accuracy) of the displacement measurement. Figure 6(b) shows the mean optical flow magnitude for the control group and all electrode insertion types. The displacement magnitude as measured by optical flow was larger for all insertion steps of all microelectrode groups compared with baseline, reaching significance for the 1- and 2-shank groups (p<0.05).

**Fig. 7:**
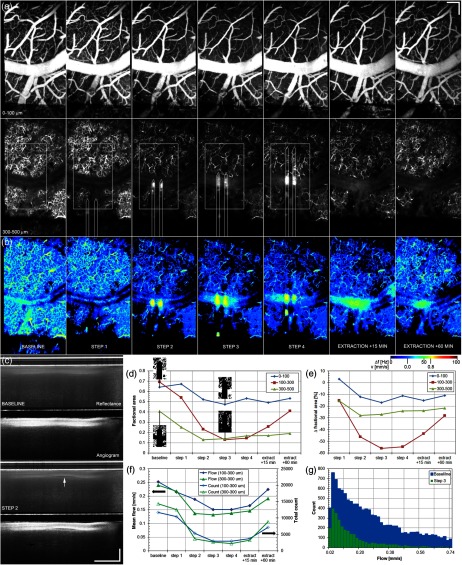
ACA branch is compressed during electrode insertion causing a reversible drop in flow. (a) Shown are the slice 1 and slice 3 angiograms for baseline, four insertion steps, and two extraction steps. Slice 3 overlays include ROI and approximate electrode locations. (b) Shown are the corresponding slice 3 flow maps. The ACA branch (large horizontal vessel) is partially compressed during step 2, causing the flow to decrease across the window at all depths. (c) Cross-sectional OCT/OCA images for baseline and step 2 showing ∼20% compression in the ACA branch (white arrow), possibly causing a drop in flow. Interestingly, the electrodes (black arrow) at this B-scan are significantly deeper than the vessel. Graphs indicate: (d) the measured perfused capillary density (factional area), (e) density change from baseline, and (f) mean and total flow (count) for ROIs around the electrodes for the sequence. (g) Individual flow histograms for slice 2 (100 to 300  μm) indicate a drop at all velocities. Scale bars=200  μm. Video 3 shows all layers and steps, including reflectance, angiography, and flow maps. (Video 3, AVI, W82, 1.5 MB) [URL: https://doi.org/10.1117/1.NPh.3.2.025002.3].

In addition to bulk mechanical tissue displacement, electrode insertion also caused potentially more pernicious consequences arising from underlying mechanical forces. Figure 7 shows images from one animal with a significant global (i.e., under the entire window) loss of vessel density and blood flow during insertion. The evidence from three-dimensional angiography datasets suggests that electrode insertion caused compression in one of the larger vessels that feeds the entire area under the window. First, the vessel density [Fig. 7(a), slices 1 and 3] and flow [Fig. 7(b)] decreases were most pronounced between the first and second insertion step, as the electrode passed under an ACA branch, the largest vessel in the field of view. Second, the density and flow began to recover in the capillary network immediately after electrode extraction. Third, cross-sectional OCT reflectance and angiography images near the center of the ACA branch suggest compression [Fig. 7(c), white arrow]. Interestingly, the electrode is well below the vessel at this point [Fig. 7(c), black arrow], suggesting that any vessel compression may have been caused by being sandwiched between the electrodes and the window. Using the cross-sectional image, the ACA branch diameter measured ∼18% smaller after insertion step 2 compared with baseline (decrease from 112 to 92  μm), but since the vessel depth plane is slightly out of focus, care must be taken in interpreting this result. Figures 7(d) and 7(e) quantitatively show the drop in vessel density with insertion and initial recovery with extraction. Although the change was largest in slice 2 (100 to 300  μm), all slices exhibited similar trends, indicating the region affected by compression of this vessel extended over a large cortical depth range >0.5  mm. Figure 7(f) shows the change in mean flow and total flow (total histogram count) and Figure 7(g) shows the flow histograms for baseline and insertion step 2, indicating full spectrum flow loss (0.0 to 0.8  mm/s). Although the most straight-forward explanation of the result is mechanical vessel compression, we cannot rule out vessel constriction induced by some aspect of electrode insertion.

### 3.3 Perfused Vessel Density

**Fig. 8:**
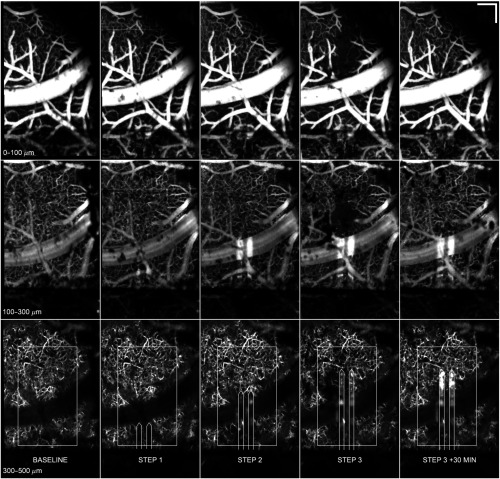
Electrode insertion caused rapid, localized flow drop-out in the region around the electrode. Shown are the slice 1 to 3 angiograms for baseline, three insertion steps, and one additional time-point (showing recovery). Flow drop-out occurs primarily in slices 1 and 2, though some decrease in slice 3 is also observed. Slice 3 overlays include ROI and approximate electrode locations. Scale bar=200  μm. Video 4 shows the sequence, including reflectance images, angiograms, and flow maps. (Video 4, AVI, W83, 1.1 MB) [URL: https://doi.org/10.1117/1.NPh.3.2.025002.4].

In one animal, there was rapid, local flow-drop-out in the region immediately surrounding the electrode without observable cause. Figure 8 shows the angiograms for all three slices for baseline, three steps, and one time-point (30 min) after the final step. The flow drop-out occurred immediately after insertion step 3 and was resolved 30 min later. The drop-out was most pronounced in slice 1 and 2 (0 to 300  μm), though it also affected slice 3 (300 to 500  μm). Though surface debris from irrigation increased slightly with steps 3 and 30 min later, cross-sectional and *en face* OCT reflectance images indicate that the drop-out was not an artifact resulting from shadowing above the window. One or two other animals had some evidence of local flow deficits, though not to the degree found with this animal. It is also possible that all animals exhibit this rapid change but that it was only captured in this animal. Because the imaging was nearly continuous for all animals, this is not likely. The cause of the flow drop-out is not known, but the pattern of drop-out suggests it is a direct result of electrode insertion.

**Fig. 9:**
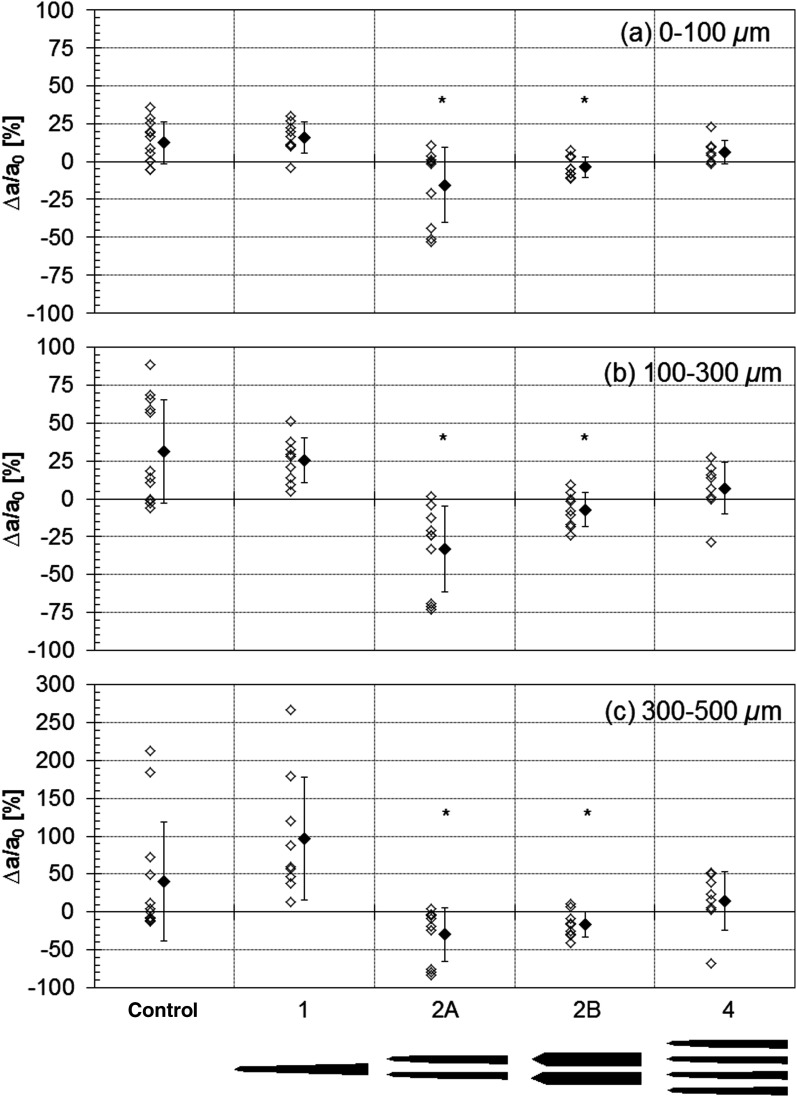
Percent change in vessel density (fractional area) from baseline for control and four electrode groups in a $1\times 0.6\;{\mathrm{mm}}^{2}$ ROI around electrodes at the three depth slices examined. In (a)–(c), individual open symbols represent a single insertion step for a single animal and closed symbols are the mean change. Error bars represent ±SD. *p<0.05 between control and electrode groups. Electrode footprints are shown below graphs.

For all animals, the perfused vessel density in the region around the inserted electrodes was measured using the fractional vessel area (sum of vessel pixels/total ROI pixels, excluding masked electrode region) for each OCA integrated depth slice. Figure 9 shows the change in vessel density from baseline for control and four electrode groups at the three depth slices examined. The three slices are represented in Figs. 9(a)–9(c), where the percent change ($\Delta a/a_{0}$) for each insertion step of each animal (open symbols) and the mean percent change for the group (closed symbols) are shown. Only the 2-shank electrode groups (A and B) had a statistically significant change from control (p<0.05).

Overall, the vessel density changes were minimal, except for the control group animal highlighted in Fig. 3 (W87), the animal that appeared to have a significant vessel compression (Fig. 7, W82), and the animal with temporary flow drop-out (Fig. 8, W83), the last two of which were 2-shank A microelectrodes. The perfused capillary density change did increase with depth, with the number of steps that exhibited a change greater than 20% increasing from 23% to 46% to 52%, from slice 1 to 2 to 3, respectively. Seventy five percent of the animals had an increase exceeding 20% in at least one step for slice 3 (300 to 500  μm). In terms of the perfused capillary density change as a function of insertion step, the change was generally larger in steps 2 and 3 than step 1 (∼2 to 3 times larger), reaching significance for slice 2 (100 to 300  μm) in the electrode animals, where the change in step 3 was 3.6 times larger than in step 1. Part of the reason for this is that the perfused capillary density analysis, unlike the optical flow analysis, was referenced to baseline for each insertion step, therefore some cumulative effect is likely to result. However, another possible explanation is that the lateral and axial extent of the interaction region between electrode and tissue is smallest for the first insertion step. The control animals showed a similar trend but, as expected, no significant differences between steps.

### 3.4 Flow Velocity

**Fig. 10:**
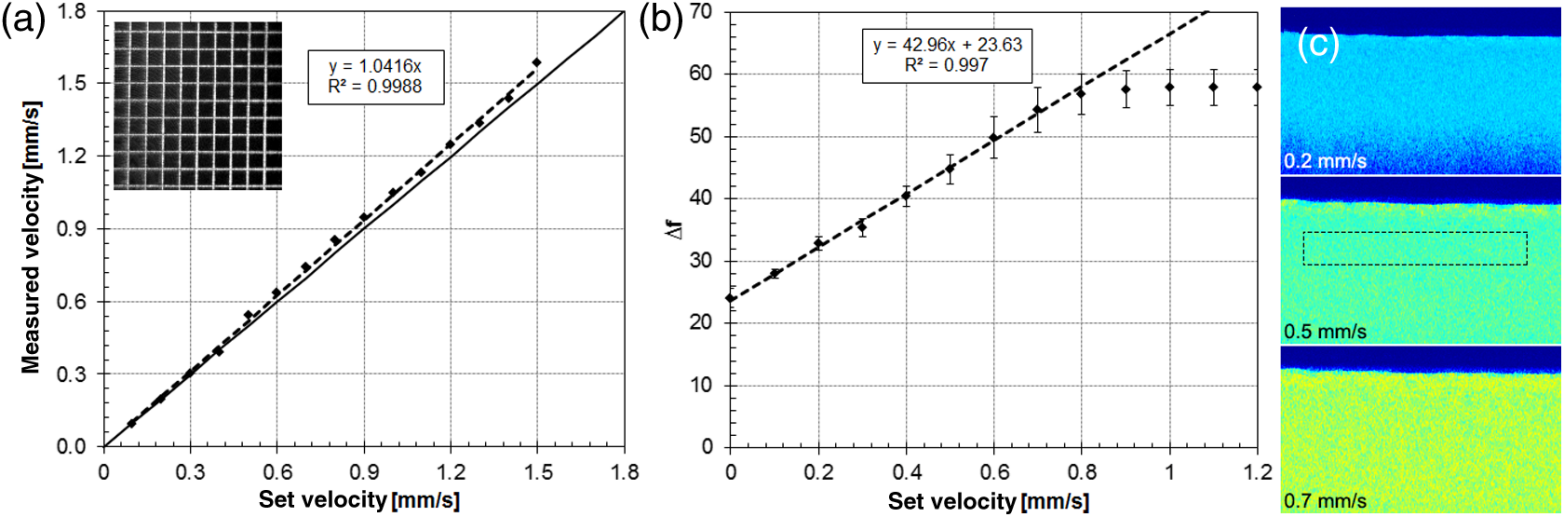
Flow calibration using grid and uniform scattering phantoms and high precision, motorized translation stage. (a) Measurements to verify accuracy of stage velocity. Dotted line is high correlation fit between set and measured stage velocity. Solid line is a slope of one. Inset shows *en face* OCT image of grid phantom. (b) Measurement to define the linear relationship between mean Δf and stage velocity. Error bars represent ±SD. Slope, intercept, and correlation coefficient for linear fit are shown. (c) OCT flow maps of the phantom at each of the speeds indicated. Dotted region is ROI from which mean±SD Δf was calculated.

In order to quantify flow velocity, it was necessary to define the relationship between the measured Δf flow map and velocity within the measurable range of the velocimetry algorithm. This was done with phantoms on a precision stage moving at a constant linear velocity. For all calibration measurements, the stage was set in motion for a few seconds before imaging to prevent acceleration or backlash transients and moved in the same direction to increase the calibration accuracy. First, the phantom was positioned so that the grid was aligned to the scan axes (i.e., with no angle between grid and scan beam) and verified with an *en face* OCT image of the square grid pattern [see inset of Fig. 10(a)]. Second, the stage accuracy was verified with a grid phantom as shown in Fig. 10(a). The grid phantom, with 100-μm line separation, was imaged at an OCT frame rate of 134 fps while the stage was set to a constant velocity. The measured velocity was found by tracking the distance traveled by individual focused dots, which represented the grid lines in the OCT B-scan image, for the acquisition duration. The stages were linear and accurate to ∼4% within the range from 0 to 1.5  mm/s. Next, the calibration curve to calculate flow velocity from the Δf flow map was found using a highly scattering phantom as shown in Fig. 10(b). The curve is predominantly dependent on the B-scan interval (4.16 ms for the flow sets). For these measurements, the B-scan interval, sigmoid transparency width, and range parameters were set identical to those used during the animal imaging sessions. The image of the phantom at three different velocities is shown in Fig. 10(c). For each data point, an ROI was selected and the mean value plotted as a function of set velocity. The linear fit to the data in the range 0.0 to 0.8  mm/s had a correlation coefficient of 0.997. The slope and intercept of this calibration curve were used to calculate the flow velocity of capillaries measured in the mouse motor cortex.

**Fig. 11:**
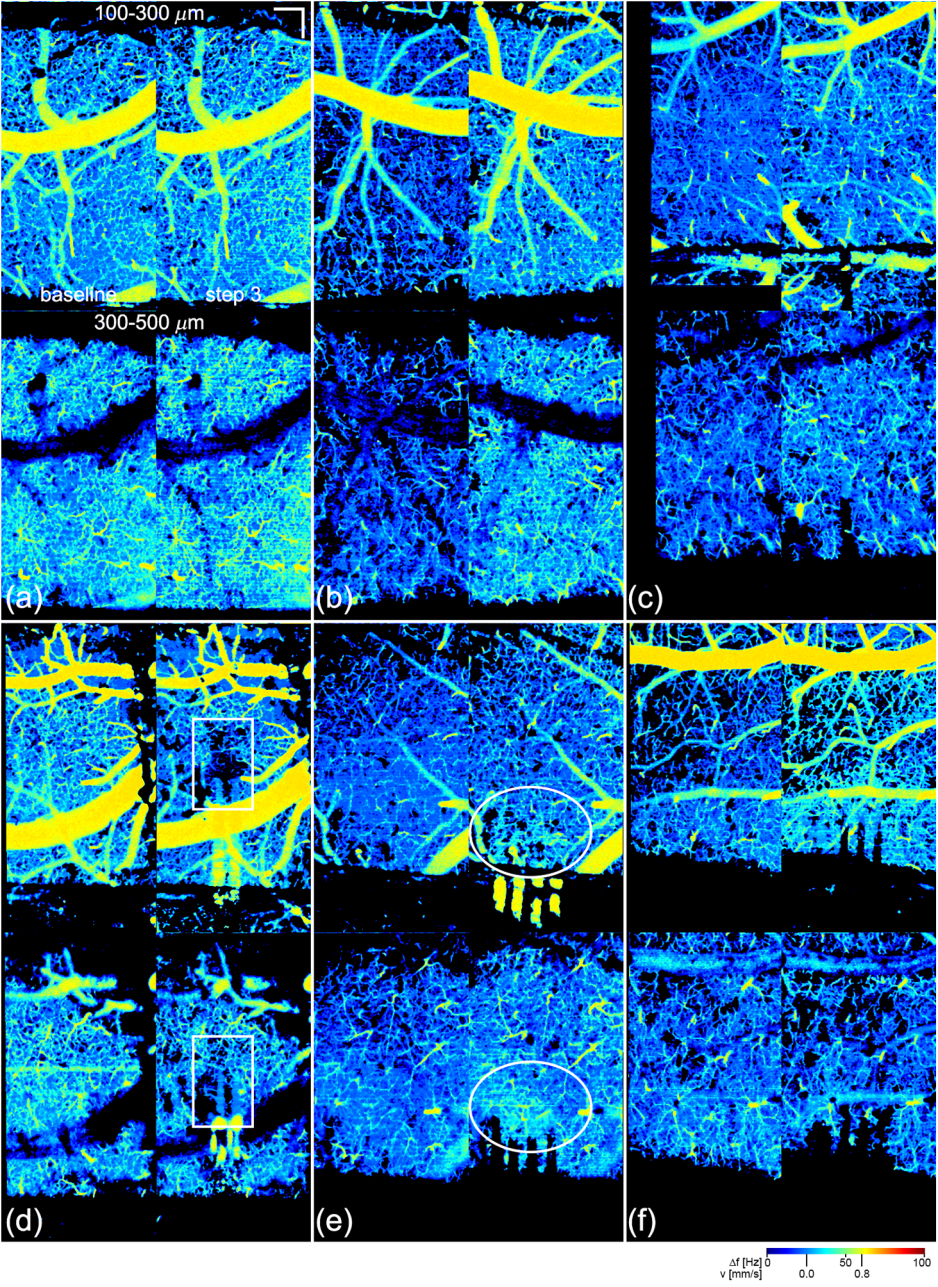
Six different capillary velocimetry examples. Shown are two control animals: (a) one with no change in flow and (b) one with an increase in flow. Four electrode animals are shown: (c) 1-shank electrode animal with moderate global flow increase, (d) 2-shank B electrode animal with a local flow drop-out (boxed ROI), (e) 4-shank electrode animal with an apparent increase in local flow in the region around the electrode (circle ROI), and (f) 4-shank electrode animal with increase in slice 2 and decrease in slice 3. For each animal, four flow maps are shown for slices 2 (upper panels) and slice 3 (lower panels) and baseline (left panels) and insertion step 3 (right panels). Scale bar=200  μm. Videos 5, 6, and 7 show the sequence, including reflectance images, angiograms, and flow maps. (Videos 5, 6, 7, AVI, W88, W87, W79, W83, W75, W77, 1.0 MB; 0.9 MB; 0.7 MB) [URL: https://doi.org/10.1117/1.NPh.3.2.025002.5]; [URL: https://doi.org/10.1117/1.NPh.3.2.025002.6]; [URL: https://doi.org/10.1117/1.NPh.3.2.025002.7].

Figure 11 shows the flow maps for six different animals (two control and four electrode) used in this study, each with notable characteristics. Shown in each panel (animal) is the baseline (left) and full insertion step 3 (right) flow map for slices 2 (upper) and 3 (lower). In the control animals, the flow and density results matched: in two animals the flow was essentially unchanged [e.g., Fig. 11(a)] while in two others there was an observable increase in flow [e.g., Fig. 11(b)]. In the electrode animals, a small increase in flow, as observed for the 1-shank animal in Fig. 11(c), cannot be entirely attributed to the insertion because of the control animal results. Notable results occurred for the electrode animals when there was a local flow decrease [e.g., Fig. 11(d)], a local flow increase [e.g., Fig. 11(e)], or a global flow decrease (not shown). For most of the animals, the flow in the two layers tracked. However, in at least one animal, shown in Fig. 11(f), the flow appeared to increase in slice 2 and decrease in slice 3.

**Fig. 12:**
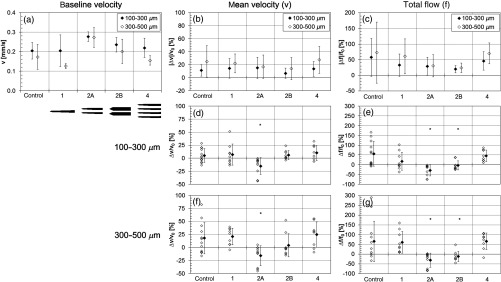
Capillary flow quantification for slice 2 and slice 3 for the flow range from 0.0 to 0.8  mm/s in a $1\times 0.6\;{\mathrm{mm}}^{2}$ ROI around electrodes. (a) Mean baseline blood flow velocity [mm/s]. Electrode footprints are shown below graph. Percent change in mean velocity (b) and total flow (c) magnitude (absolute value) from baseline averaged over all insertion steps and all animals. Percent change in mean velocity (d) and (f) and total flow (e) and (g) for all animals and all insertion steps. The total flow is the total ROI histogram count, accounting for ROI size, which varied for each electrode animal. For (d)–(g), individual open symbols represent a single insertion step for a single animal and closed symbols are the mean change. Error bars represent ±SD. *p<0.05 between control and electrode groups.

The flow velocity measurements for all animals for slices 2 and 3 are shown in Fig. 12. The mean baseline flow velocity is shown in Fig. 12(a). For all animals the mean baseline velocity in slices 2 and 3 was 0.23 (0.05 SD) and 0.18 (0.07 SD) mm/s. It is noted that flow was observed in capillaries across the entire 0 to 0.8  mm/s range in each ROI [see histogram in Fig. 7(g)]. The mean velocity (v) and total flow (f) are shown in the next two columns. Figures 12(b) and 12(c) show the magnitude (absolute value) of the flow changes for both slices ($\left|\Delta v\right|/v_{0}$ and $\left|\Delta f\right|/f_{0}$). The percent change in velocity from baseline ($\Delta v/v_{0}$) for each step is shown for both slices [Figs. 12(d) and 12(f)]. The percent change in total flow [Figs. 12(e) and 12(g)] was found by first calculating the percentage of flow pixels in the ROI within the 0.0 to 0.8  mm/s range (sum of flow pixels/total ROI pixels, excluding masked electrode region) and then calculating the change from baseline ($\Delta f/f_{0}$). Like Fig. 9, the open symbols represent the percent change ($\Delta v/v_{0}$ and $\Delta f/f_{0}$), while the closed symbols are the mean percent change for all animals in the group.

Both mean velocity and total flow measurements show similar trends for the groups, with the mean velocity exhibiting a much lower magnitude of change, likely because flow outside of the measurement range has already been excluded [see histogram in Fig. 7(g)]. The only statistically significant differences (p<0.05) in electrode groups compared to the control group was the 2 shank A group for mean velocity and both 2-shank groups (A and B) for the total flow. There were no statistical differences in flow change magnitude for any electrode group compared with the control group. In terms of the blood flow change as a function of insertion step, like the perfused capillary density results, for the electrode groups, step 1 had a lower magnitude change than the other steps, likely for the same reason stated above. The control animals showed no significant blood flow differences between insertion steps. The decrease in blood flow in many of the 2-shank electrode animals mirrored that found in the density measurement, which is expected, considering the fact that the angiogram and flow measurement both derive from the same physiological phenomena, i.e., flowing blood.

## 4 Discussion

Our previous longitudinal imaging study of neural implants showed new vessel growth and remodeling in superficial cortical layers associated with craniotomy and window insertion over the course of weeks to months after surgery, and no change or only subtle change in the deeper capillary network surrounding the electrode.^29^ In this study, we sought to investigate any immediate changes in vasculature and flow during electrode insertion with quantitative optical coherence angiography (qOCA). We did not expect angiogenesis or capillary remodeling over the acute time-course (2 to 3 h) of this study, and indeed we observed no new capillary growth or migration of existing capillaries.

The control animal results clearly indicate both the large variability in basal flow in individual animals (compare left panels of Figs. 3 and 4) and that the window surgery itself can result in relatively immediate flow changes in the region exposed by craniotomy (Fig. 4). Isoflurane is known to cause vasodilation^37^ and could contribute to increased flow. However, any affect from the anesthesia is assumed to be smaller than the surgery itself because the animals are maintained on a constant dose through-out the surgery, which takes about 2 h, and imaging, which takes another 2 to 3 h. Regardless of the cause, some control animals without implanted electrode exhibited an increase in capillary perfusion within hours after craniotomy/window insertion. No decrease in flow was observed in any control animal in this study or our previous results.^29^ Therefore, it is reasonable to assume that any decrease in perfused capillary density or flow velocity is the result of electrode insertion.

The primary effect of microelectrode insertion observed in the results was related to mechanical trauma. In one case (Fig. 5), an arteriole was punctured during insertion, causing immediate disruption of the blood-brain barrier. It appears that the perfused capillary density had already begun to increase at the time the vessel was ruptured, and so it is difficult to determine if the further increase in density in subsequent steps was due to localized trauma or global trauma from the craniotomy. In another case (Fig. 7), the electrodes compressed the ACA branch that supplies the region, causing a global drop in flow (perfused capillary density) and velocity. Density and flow rates returned when the electrode was extracted. The cross-sections show that the electrode is 200  μm below the vessel at this insertion step, indicating the entire parenchyma between the window and electrode was involved in the compression. This result indicates the possibility for insertion-induced hypoxia even without vessel rupture. While the window is part of the experimental apparatus and not a clinical consideration, similar forces could be applied between shanks of a microelectrode array or between an electrode and the skull or plates inserted in the skull to seal the surgical field. The microelectrodes in this study were necessarily inserted at an angle for visualization below the window, whereas in the clinical case they would generally be inserted perpendicular to the cortical surface. The anatomy of cortical vasculature includes larger superficial vessels, pial capillaries, and perpendicular penetrating arterioles and venules. Mechanical trauma for any of these vessel types is not expected to be particularly sensitive to insertion angle. For all electrode types, the optical flow measurements indicate some degree of mechanical disruption, including displacements of tissue up to ∼35  μm due to electrode insertion. The vessel displacement measured with optical flow was largest for the 2-shank electrode types, though this was due more to the proximity of the large ACA branch to the insertion point for these surgeries. Optical flow is a straight-forward way to visualize and quantify displacement across the entire field of view, and was enhanced when applied to angiography images compared with reflectance images. Optical flow analysis as applied to integrated depth slices measures only lateral mechanical motion, and axial compression/expansion would require further analysis on the OCT cross-sections.

There were no clear trends between microelectrode array footprint in the perfused capillary density and flow velocity results (Figs. 9 and 12), though the 2-shank electrode exhibited the largest decrease in perfused capillary density and flow velocity. Like the mechanical response measured by optical flow, the 2-shank decrease is probably due mostly to the relative position of the ACA branch to the electrode insertion point under the window. The proximity of a large vessel to the electrode insertion point and the possibility that a large vessel could become compressed or partially occluded when pressed between adjacent electrodes in an array are conditions that can be considered by surgeons and electrode designers to minimize trauma and ischemia. The most interesting results may be the local flow changes that occurred. In a few animals, as visualized most clearly in Fig. 8, there was a local flow drop-out in the immediate 100 to 200-μm region surrounding the electrode. The cause is not obvious and could have been mechanical (occlusion of a smaller vessel), though the pattern of the drop-out indicates a reaction to the electrodes themselves and capillary microrupture cannot be ruled out. The flow drop-out occurred in all three slices but was more extensive in the superficial slices, so compression of a penetrating arteriole is also a possible cause.

The results presented herein seem to confirm some of the observations of Kozai et al.,^28^ who describe the extension of microglia processes but no mass migration of microglia toward implanted microelectrodes in the acute implantation phase. Such a migration would probably elicit a higher metabolic demand, which presumably would be sensed in our study. Kozai et al. also observed tissue displacement, though the movement was after electrode implantation. We observed rupture of a larger pial vessel during electrode insertion in one animal but no obvious microhemorrhages in the capillary network similar to that reported by Kozai et al., though the lateral resolution was considerably higher in their TPM imaging field of view (∼0.4 versus 2  μm). Also, pooled blood is not detectable by the amplitude decorrelation technique, though if the extravasation volume is large enough, OCT signal shadowing of deeper structures can occur. In our own initial histological studies,^27^ some differences in electrode array footprint have been observed, though that study used blackrock-type arrays, which are ill suited to imaging studies because of their arrangement of electrodes and opaque substrate. While the present study sheds light on the acute vascular dynamics, further investigations are required to quantify the tissue response over the entire temporal course of electrode implantation, from initial insertion through device failure.

Our work provides data only on the changes in vascular perfusion and flow during electrode insertion, and does not measure the effect on neural signaling. However, alterations in vascular dynamics that occur during surgical procedures involving electrode insertion can affect neural activity and the subsequent performance of implanted electrodes. Generally, disruptions in vascular perfusion, such as those seen in cortical spreading depression,^38^ reduce spontaneous neural activity through effects on pre- and postsynaptic mechanisms.^39^ Following electrode insertion, extravasion of blood into the surrounding parenchyma is associated with reduced recording electrode performance.^40^ Results from the same study also demonstrate that bleeding during surgical procedures alone is sufficient to cause tissue damage and atrophy, which presumably lead to electrode performance degradation. In addition, local infiltration of blood-borne macrophages plays a primary role in mediating neuroinflammation following electrode insertion, and is correlated with neurodegeneration.^41^ While the association is indirect, these studies provide strong evidence that vascular perfusion and injury are intimately related to neuronal health, and in turn, the efficacy of implanted recording electrodes. Future studies will incorporate simultaneous electrophysiological recording with OCT and TPM imaging to provide a direct link between vascular dynamics and neural activity during electrode implantation.

### 4.1 Limitations of Current Study

qOCA is a powerful label-free tool for neuroscience that can complement the information collected from other imaging modalities, especially TPM, for studies of blood flow. The source of contrast is intrinsic scatter from moving erythrocytes and so no dye injection is required. As shown in this paper, the technique allows relatively rapid acquisition of angiography images and quantification of global vascular or blood flow changes across an entire field. However, there are some limitations in qOCA techniques generally, and in this study in particular. First, qOCA is generally insensitive to pooled (static) blood, other than changes in OCT reflectance from blood absorption (i.e., shadowing), and so is less suited to detect extravasation than dye angiography. Second, capillary velocimetry by qOCA generally requires long integration times of several minutes for volumetric datasets. This is due to lower flow rates, but also to obtain optimal SNR flow maps. Velocimetry of penetrating arterioles can be accomplished rapidly using quantitative Doppler techniques^22^^,^^23^ because of the small angle between illumination beam and vessel. Capillaries are nearly perpendicular to the beam and so are ill suited to Doppler techniques. OCA capillary velocimetry estimates, like any flow estimates derived from optical images, are susceptible to inaccuracy with respect to the back-scattered signal intensity, especially for deeper vessels.

There were other design constraints and confounds to interpretation of results in this investigation. An adequate working distance under the microscope objective for electrode insertion limited the lateral resolution, though in this case capillaries were resolved to a depth of 500 μm in the cortex under the window. Optimal choice of gate length (i.e., consecutive B-scans) for SNR,^31^ as well as the inherent averaging of the autocorrelation function in the flow algorithm enhanced the ability to resolve capillaries with a 6.5-μm spot. Because the tissue exposed after surgery for electrode insertion required irrigation, in some instances residue was left on the window and could shadow the structures beneath. The preparation is unique to this acute insertion study, as a longitudinal study preparation would involve sealing the window and electrode to (relatively) fixed positions with respect to the cortex. We were careful to check for residue and shadowing in the cross-sectional images, which manifest as a reflectance differential, whenever there were changes in vascular structure or flow.

Finally, subject variability made interpretation of results especially difficult. Careful surgical procedures minimized but did not eliminate extravasation from craniotomy. The vascular network under the window was also quite varied from animal to animal. Most importantly however, the response to the craniotomy surgery and window placement varied across the both the experimental and control cohorts. Quantification of the dynamics of a more subtle vascular response to electrode implantation deeper within the capillary network in the presence of a global vasodilatory response to craniotomy was difficult.

## 5 Conclusion

qOCA uses intrinsic contrast to provide a wealth of information on vascular dynamics for a variety of applications in neuroscience. Here we used it to quantify the tissue displacement (via optical flow), change in perfused capillary density, and change in capillary flow velocity during penetration microelectrode insertion into mouse motor cortex. Innovative enhancements to OCT, particularly deeper tissue penetration to the corpus collosum with vertical cavity surface emitting laser (VCSEL) technology^42^ and longer wavelength (1.7  μm) sources ^43^ should lead to new discoveries of cortical neurovascular dynamics and especially the interplay between brain and machine. Our future investigations will focus on neuro-vascular interaction with TPM and OCA, pial-arteriole relationship with capillary velocimetry and Doppler imaging, combined imaging and electrophysiology, and cortical stimulation.
